# Secrecy Performance Analysis of Wireless Powered Sensor Networks Under Saturation Nonlinear Energy Harvesting and Activation Threshold

**DOI:** 10.3390/s20061632

**Published:** 2020-03-14

**Authors:** Xiaohui Shang, Hao Yin, Yida Wang, Mu Li, Yong Wang

**Affiliations:** 1 College of Communications Engineering, Army Engineering University of PLA, Nanjing 210007, China; dadawang333@sina.com (Y.W.); wangyong_85@126.com (Y.W.); 2 Institute of Systems Engineering, AMS, Beijing 100039, China; yinhao10201214@163.com; 3 61618 Troops of PLA, Beijing 100094, China; lmson3690@sina.com

**Keywords:** wireless powered sensor networks, nonlinear energy harvesting, activation threshold, generalized multiuser scheduling, secrecy outage probability, physical layer security

## Abstract

In this paper, we investigate the impact of saturation nonlinear energy harvesting (EH) and activation threshold on the multiuser wireless powered sensor networks (WPSNs) from the physical layer security (PLS) perspective. In particular, for improving the secrecy performance, the generalized multiuser scheduling (GMS) scheme is exploited, in which the *K*th strongest sensor is chosen based on the legitimate link. For evaluating the impact of various key parameters on the security of system, we obtain the exact closed-form expressions for secrecy outage probability (SOP) under linear EH (LEH), saturation nonlinear EH (SNEH) and saturation nonlinear EH with activation threshold (SNAT), respectively, and solve the maximization problem of secure energy efficiency (SEE). Simulation results demonstrate that: (1) the number of source sensors, the EH efficiency and the transmit power of power beacon (PB) all have positive impact on SOP, and the smaller generalized selection coefficient is advantageous for secrecy performance; (2) LEH is an ideal situation for SNEH when the saturation threshold is large enough and SNEH is a special situation for SNAT when the activation threshold is low enough; (3) the time-switching factor and the activation threshold both have an important impact on the secrecy performance, which are worth considering carefully.

## 1. Introduction

The Internet of Things (IoT), as the intelligent infrastructure in the fifth-generation (5G) mobile communication networks, has been popularized and applied all over the world in recent years [1,2,3]. It plays a remarkable role in all aspects of our daily lives and can be deployed in many fields, including healthcare, automobile, industrial appliances, sports, homes, and so on. Billions of small “things” talk to each other and cooperate to fulfill some sophisticated tasks, which not only arouse exciting opportunities for economic growth, but also opens the door to a variety of new security threats. However, restricted by the objective demand of low cost and lightweight, the devices in IoT, especially in wireless sensor networks (WSNs) [4,5], which are considered to be the main application scenario of the future IoT, are generally characterized by limited resources, battery power supply, low energy consumption and poor computing capacity. The rapid development of wireless communication will give rise to the massive deployment of sensors nodes and a vast amount of information exchange, making it unfeasible to flexibly recharge and control the power-constricted devices. Therefore, energy shortage is one of the biggest challenges restricting the effective and widespread application of WSNs and IoT [6]. Due to the rising power costs, green-oriented methods have inevitably become the dominating design consideration in the WSNs system. Fortunately, wireless powered communication (WPC) technology based on the far field radio frequency (RF) energy harvesting (EH), as a promising paradigm to tackle the energy barrier suffered by traditional wireless communication networks as well as emerging WSNs, has been attracting worldwide attention by the researchers in recent years [7,8,9,10,11,12]. In contrast to traditional wireless sensors, which rely on batteries or are powered by wired charging for their energy need, the devices in wireless powered sensor networks (WPSNs) can be able to harvest energy from RF signals wirelessly. Consequently, it can fundamentally release the heavy burden caused by frequent battery replacement and/or recharging, avoid the communication interruption caused by energy loss, and ensure theoretical permanent life for communication systems [13]. A basic hurdle of WPSNs rest with the fast attenuation of radio frequency (RF) signals with distance changing. Moreover, energy amount harvested at wireless powered devices is fairly restricted, which limits the coverage severely and has become a bottleneck for the widespread application of WPSNs. Consequently, the design of an energy-efficient transmission is of great significance to introduce the green concept into future WPSNs.

Meanwhile, compared with the traditional WSNs, both the topological structure and the radio environment of WPSNs are more complex and the nodes are more diverse, which makes information interaction face more severe security threats [12,13]. Typically, the algorithms based on the upper layer encryption mechanism are explored to ensure information security in wireless communication. Unfortunately, with the continuous development of computer technologies and the emergence of quantum computers, the conventional cryptographic techniques can no longer satisfy the requirements for confidentiality of wireless transmissions. In addition, the tedious algorithms are restricted for many lightweight sensors owing to requirements of high hardware complexity and more energy. Therefore, for the further enhancement of information security, physical layer security (PLS), which is a promising way to ensure the security of wireless networks, has been considered an effective supplement for the existing solutions [14,15,16]. With regard to the wireless PLS, its basic idea is to make use of the characteristics of randomness and openness of wireless channels to reliably transmit information from legitimate transmitters to expected receivers, and to ensure that confidential information is not intercepted or eavesdropped [15]. To improve the secrecy performance of wireless communication network effectively, the researchers have carried out a lot of approaches to expand the difference between the legitimate channel and the eavesdropping channel. At present, the widely used enhancement technologies of PLS can be divided into several categories [17]: physical layer secret key generation, coding-based secure transmission, security-oriented beamforming; artificial noise (AN) assisted technology and security diversity technology, etc.

Recently, many works have investigated the PLS in cognitive radio networks (CRNs) [18,19] and in WPSNs [20,21,22,23,24,25,26,27,28,29,30,31,32,33]. Specifically, the authors in [18] analyze a secure dual-hop mixed radio frequency-free space optical (RF-FSO) downlink simultaneous wireless information and power transfer system. The authors in [19] consider an underlay multiple-input-multiple-output (MIMO) CRN. On the other hand, aimed at direct communication scenarios, the authors in [22] investigated the secure beamforming schemes in a multi-input single-output (MISO) downlink system with a multi-antenna transmitter and multiple energy receivers (ERs). Secrecy performance for a single-input multi-output (SIMO) wireless powered network is presented in [23]. Later, in [24], the system model in [23] was extended to a wireless powered MIMO network. For further improving the security of WPSNs, Refs. [25,26,27] explored the rational use of wireless powered friendly jammer, where the jammer harvests wireless energy from external RF signal before sending the artificial noise (AN) to confuse eavesdroppers. On the other hand, for the dual-hop cooperative communication scenarios, the cooperative jamming (CJ) scheme and the zero-forcing beamforming was designed to enhance the security of WPSNs in [28,29,30]. With the help of relay-selection strategy, [31,32,33] investigated secure communication in multiple relays aided WPSNs. In particular, ref. [33] designed a secure energy-efficient (SEE) transmission strategy for wireless powered IoT with multiple power beacons (PBs), which can provide a stable energy supply for the WPSNs flexibly. It is worth remarking that most of works pay much more attention to the improvement of secrecy performance contributed by multi-antenna diversity, cooperation diversity and AN-aided method, but a few pay attention to the practical scenario with multiuser scheduling [34,35], which can be seen as the typical application of future WSNs.

On the other hand, a major limitation of aforementioned works is that they all considered the linear mode for energy harvester, which has the advantage of easy analysis and processing. However, as a matter of fact, the practical EH circuits usually show a nonlinear feature rather than a linear one. Compared to the nonlinear EH model, the linear one may lead to the significant performance degradation in practical WPSNs. In particular, the recent advanced works in [36,37,38] pointed out that the nonlinear EH mode is more reasonable due to the power conversion circuits, capacitors, inductors, and diodes used in the electronic devices are nonlinear commonly. The over-simplified linear EH modes do not capture the nonlinear characteristics of the rectenna accurately enough and may lead to severe resource allocation mismatches, resulting in the performance to drop significantly in practical applications [11,39]. Furthermore, the authors in [40] introduced that the saturation nonlinear mode is closer to the experimental results derived from the actual RF-based EH devices. Recently, although several works have investigated the impacts of the saturation nonlinear EH mode on the secrecy performance of WPSNs [36,39,41], the considered models are too complex and the computational complexity of the proposed schemes is too high, which is unhelpful to extract deep insights of the practical saturation nonlinearity. Moreover, the exact closed-form expression of secrecy outage probability (SOP), which is regarded as an important indicator of secrecy performance, has been not given in [36]. Additionally, it is necessary to explore the effect of activation threshold of the power conversion circuits on the security of WPSNs. The so-called activation threshold means that when the input power of the EH circuit is lower than the threshold value, it will always be inactive, resulting in the device unable to transmit information. On the contrary, it can turn on the energy harvester and provide continuous power for the normal transmission of information [33,38]. Specifically, for the considered WPSNs, the nonlinearity of EH has an important impact on the secrecy performance of the system. Therefore, to expose the trade-off between the energy consumption and the security, SEE is a proper metric to indicate this relationship. Although there are lots of papers about the secure transmission or energy-saving in WPSNs, they always ignore this trade-off.

Enlightened by previous observations, this paper investigates the impact of nonlinear EH and activation threshold on the multiuser WPSNs with generalized multiuser scheduling (GMS) from the PLS perspective, where multiple source sensors that perform monitoring or operating tasks in the local group harvest energy from the dedicated PB in the presence of an external eavesdropper. In particular, the GMS scheme is used at the source sensors based on the legitimate link to improve the secrecy performance. Moreover, by comparing with general linear EH mode, we deeply analyze the influence of nonlinear EH circuit and energy activation threshold on the secrecy performance of WPSNs. The main contributions of this paper are as below:

- We explore the PLS in the multiuser WPSNs with GMS scheme, where the *K*th-best source sensor is chosen by the main channel for improving the security. Furthermore, we obtain the exact closed expressions for SOP of the system under linear energy harvesting (LEH), saturation nonlinear energy harvesting (SNEH) and saturation nonlinear energy harvesting with activation threshold (SNAT), respectively.
- With the help of the SOP expressions, we further discuss the impact of various key parameters on secrecy performance of the multiuser WPSNs, including the number of source sensor, the EH efficiency factor, the generalized selection coefficient, the saturation threshold, the activation threshold, the transmit power of PB and the time-switching coefficient. To get a deeper insight, we further investigate the maximization problem of SEE and compare the SEE under three different EH modes.
- Simulation results demonstrate that increasing the number of source sensors, enhancing the EH efficiency and strengthening the transmit power of PB all favorable to improve secrecy performance of the multiuser WPSNs. Meanwhile, the smaller generalized selection coefficient is beneficial for SOP in three EH modes. Furthermore, the SOP of SNEH is better when the saturation threshold is higher and LEH is an ideal situation for SNEH when the saturation threshold is large enough. The SOP of SNAT is better when the activation threshold is lower and SNEH is a special situation for SNAT when the activation threshold is low enough. In addition, for providing secure and efficient communication, the time-switching factor should be optimized seriously. Finally, the activation threshold has an important impact on the SEE of the considered system, which is worth considering carefully.

The remainder of this paper is arranged as follows. We provide details including the system model, the process of wireless power transfer (WPT) and signal analysis, the GMS scheme and three EH modes in Section 2. Section 3 analyzes the secrecy performance of the considered model, derives the exact SOP under three different EH modes and solves the optimization problem of SEE. In Section 4, numerical results and the corresponding discussions are presented. Finally, Section 5 presents the conclusion of this paper.

## 2. System Model and Signal Analysis

### 2.1. System Model

**Figure 1:**
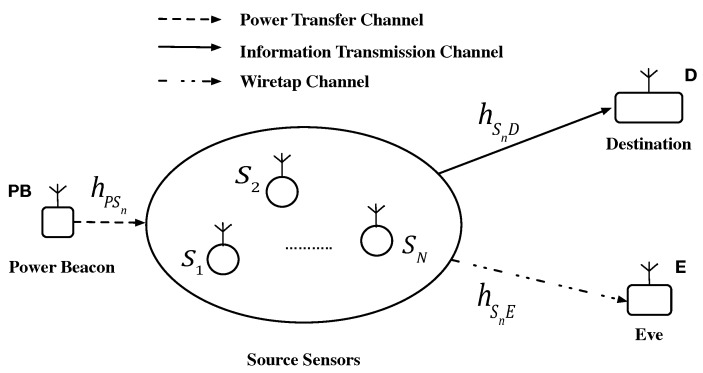
System model.

An uplink transmission in the multiuser WPSNs is considered to be illustrated in Figure 1, which consists of a dedicated power beacon (PB), multiple source sensors $S_{n}$, n∈N={1,⋯,N}, an intended destination *D*, and an eavesdropper (Eve) *E*. Due to the limitation of terminal devices, $S_{n}$ ought to acquire energy by WPT from the dedicated PB to support information transmission. In contrast, the destination *D* is powered by on-grid power. Additionally, we assume that each sensor $S_{n}$, the destination *D* and the Eve *E* are single-antenna and half-duplex devices [42]. It is worth noting that the model has numerous practical applications, such as in IoT and in low power wide area network (WAN), where the multiple sensors that restricted to a single antenna owing to cost and size limitations upload information to the access point (AP) or the base station (BS) directly.

Furthermore, in this paper, all the channels experience Rayleigh fading, which has been commonly considered to be the special case of Rician fading under the situation that the line-of-sight component is assumed to be zero. Meanwhile, it is usually adopted in works that investigating the secrecy performance of WPSNs [31,32,33,43], because it can be seen as the foundation for exploring the more practical Rician fading circumstance. It is considered that the additive white Gaussian noise (AWGN) at destination has zero mean and variance $N_{0}$. In addition, compared with the full channel situation information (CSI) assumption in [32,34], we assume only the statistic CSI is available in the passive wiretap scenario, which is more reasonable because the weak computation ability and small memory of the lightweight devices in the future wireless communication network [33]. In practice, wireless communication environments are very complex and changeable, resulting in the full CSI is hard to acquire.

For the convenience of mathematical modeling, the channel coefficients of the $P\to S_{n}$, $S_{n}\to D$ and $S_{n}\to E$ links are denoted by $h_{PS_{n}}$, $h_{S_{n}D}$, $h_{S_{n}E}$, respectively, which are considered to be subject to independent quasi-static Rayleigh fading. Furthermore, we consider that multiple source users are close in proximity, i.e., they are assumed to form a cluster. This consideration is often exploited in the investigation on WPSNs [32], which brings about the equivalent average link power gains of the channel $P\to S_{n}$, $S_{n}\to D$ and $S_{n}\to E$, respectively. For convenience, we define $\lambda_{PS_{n}}=\lambda_{PS}$, $\lambda_{S_{n}D}=\lambda_{SD}$ and $\lambda_{S_{n}E}=\lambda_{SE}$ for any n∈N.

### 2.2. Wireless Power Transfer and Signal Analysis

For WPT, the receiver in the terminal adopts EH mode based on rectangular antenna structure [44]. Specifically, the authors in [44] explore the rectenna nonlinearity from a stochastic geometry point of view. Furthermore, with regard to the rectangular rectenna, the RF signal will convert into the direct current (DC) signal through a rectifier that was composed of the passive low-pass filter (LPF) and the Schottky diode [45]. Then, it is considered that for all users, the harvested energy at the stage of WPT is entirely used for data transmission in wireless information transmission (WIT), i.e., the so-called harvest-use (HU) mode introduced as in [43] is adopted.

**Figure 2:**
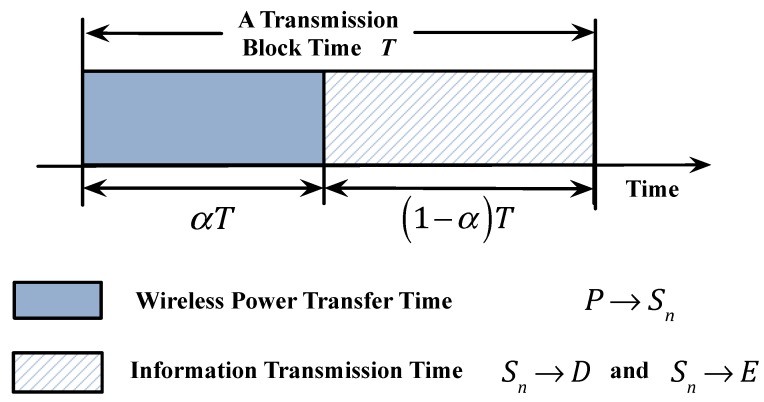
Time-switching scheme.

If the link fading factors keep unchanged within a transmission time slot *T*, one HU period including two time windows can be shown as in Figure 2. In the first phase that is denoted by αT, where α∈(0,1) is the time-switching factor, the multiple sources harvest energy from broadcast RF signal transmitted by the PB. In addition, another time window (1−α)T represents the second phase, in which a *K*th-best sensor is scheduled from multiple source sensors based on the channel quality of legitimate link (i.e., the link $S_{n}\to D$) to upload data for improving the secrecy performance, and the *E* can also interpret the message transmitted from the selected sensor. Then, the harvested energy at $S_{n}$ can be shown as below [33,46]:

(1) $$E_{S_{n}}=\eta P_{B}\alpha T{\left|h_{PS_{n}}\right|}^{2},$$

where 10%<η<80% denotes the EH efficiency factor, which mainly determined by the EH circuitry and frequencies [47]. Specifically, the relationship between the rectenna’s RF-to-DC conversion efficiency and the frequency is discussed in detail in [48]; $P_{B}$ denotes the transmit power of PB; ${\left|h_{PS_{n}}\right|}^{2}$ is power gains of the channels from the PB to $S_{n}$. It should be emphasized that the sources in WPSNs are passive sensors whose received noise power is far less than the received power contributed by PB [49]. Therefore, the harvested energy from noise is negligible.

To be specific, assume that the $n^{*}$-th sensor, which is denoted by $S_{n^{*}}$, is selected based on the GMS scheme as the information transmitting node during the latter phase. Then, the received signals at destination *D* and eavesdropper *E* are respectively expressed as

(2) $$y_{D}=\sqrt{P_{S_{n^{*}}}}h_{S_{n^{*}}D}x+n_{D},$$

(3) $$y_{E}=\sqrt{P_{S_{n^{*}}}}h_{S_{n^{*}}E}x+n_{E},$$

where *x* denotes the confidential data transmitted by the selected sensor user, $P_{S_{n^{*}}}$ represents the transmit power of $S_{n^{*}}$, $h_{S_{n^{*}}D}∼CN\left(0,\lambda_{SD}\right)$ is the channel fading coefficients between $S_{n^{*}}$ and *D*, and $h_{S_{n^{*}}E}∼CN\left(0,\lambda_{SE}\right)$ is the channel fading coefficient between $S_{n^{*}}$ and *E*. $n_{D}∼CN\left(0,N_{0}\right)$ and $n_{E}∼CN\left(0,N_{0}\right)$ are AWGN at *D* and *E*, respectively,

Furthermore, the SNRs at *D* and *E* are represented as $\gamma_{S_{n^{*}}D}$ and $\gamma_{S_{n^{*}}E}$, respectively, which can be expressed as

(4) $$\gamma_{S_{n^{*}}D}=\frac{P_{S_{n^{*}}}{\left|h_{S_{n^{*}}D}\right|}^{2}}{N_{0}},$$

(5) $$\gamma_{S_{n^{*}}E}=\frac{P_{S_{n^{*}}}{\left|h_{S_{n^{*}}E}\right|}^{2}}{N_{0}},$$

where $\;{\left|h_{S_{n^{*}}D}\right|}^{2}$ and $\;{\left|h_{S_{n^{*}}E}\right|}^{2}$ are power gains of the channels from the $S_{n^{*}}$ to *D* and $S_{n^{*}}$ to *E*.

### 2.3. Generalized Multiuser Scheduling

As described above, the Eve is a passive wiretapping node, i.e., the full CSI of Eve is unavailable. Hence, with the help of the GMS scheme, we select the sensor $S_{n^{*}}$ based on the legitimate channel only, the index of certain sensor is given as

(6) $$n^{*}=K^{th}\underset{n\in \{1,\cdots ,N\}}{\arg\max}\left({\left|h_{S_{n}D}\right|}^{2}\right),1⩽K⩽N,$$

where $K^{th}\underset{}{\arg\max}\left(\cdot \right)$ denotes the function used to decide the *K*th strongest sensor, *K* is the generalized selection coefficient and *N* is the number of source sensor.

**Lemma** **1.** *If $X_{n}$, n∈N={1⋯N}, is the random variable with the independent and identical exponential distribution (i.i.d.), the probability density function (PDF) of $X=k^{th}\max_{}\left(X_{n}\right)$ can be given by [50,51]:*

(7) $$\begin{array}{c} f_{X}\left(x\right)=\sum_{n=1}^{N-K}{\left(-1\right)}^{n}\left(\begin{array}{c} N-K\\ n \end{array}\right)\left(\begin{array}{c} N\\ K \end{array}\right)K\lambda_{X}\\ \;\;\;\;\;\;\;\;\;\times \exp\left\{-\left(n+K\right)\lambda_{X}x\right\} \end{array},$$

*where x denotes the random variable of PDF, K is the generalized selection coefficient.*

In line with the Lemma 1, the PDF of ${\left|h_{S_{n^{*}}D}\right|}^{2}$ can be given as:

(8) $$\begin{array}{c} f_{{\left|h_{S_{n^{*}}D}\right|}^{2}}\left(x\right)=\sum_{n=1}^{N-K}{\left(-1\right)}^{n}\left(\begin{array}{c} N-K\\ n \end{array}\right)\left(\begin{array}{c} N\\ K \end{array}\right)K\lambda_{SD}\\ \;\;\;\;\;\;\;\;\;\;\;\;\;\;\;\;\times \exp\left\{-\left(n+K\right)\lambda_{SD}x\right\} \end{array},$$

where $\frac{1}{\lambda_{SD}}=E\left[{\left|h_{S_{n^{*}}D}\right|}^{2}\right]$, and $E\left[\cdot \right]$ is an expectation operator.

On the other hand, the *K*th-best sensor is scheduled based on the main channel, which is independent of the wiretapping channel and corresponds to a random sensor for Eve. Consequently, the PDF of ${\left|h_{S_{n^{*}}E}\right|}^{2}$ is exponentially distributed with parameters $\frac{1}{\lambda_{SE}}$, which is shown as:

(9) $$f_{{\left|h_{S_{n^{*}}E}\right|}^{2}}\left(x\right)=\lambda_{SE}e^{-\lambda_{SE}x},$$

where $\frac{1}{\lambda_{SE}}=E\left[{\left|h_{S_{n^{*}}E}\right|}^{2}\right]$.

### 2.4. Energy Harvesting Mode

In this part, we introduce three different EH modes, i.e., linear EH (LEH), saturation nonlinear EH (SNEH) and saturation nonlinear EH with activation threshold (SNAT), respectively. In the LEH mode, the output power of the source sensor is proportional to the harvested energy, which means that the relationship between the transmit power of the sensor and the harvested energy is linear. In comparison, the transmit power of the sensor is limited by the saturation threshold in the SNEH mode. On the other hand, when considering the SNAT mode, the transmit power of the sensor is affected by both the saturation threshold and the activation threshold.

#### 2.4.1. Linear Energy Harvesting Mode

LEH mode has been widely used in a large number of previous works contributed by the intuitive and concise expression. According to the [31,32,33] and with the help of (1), under the LEH mode the output power of certain $S_{n}$, i.e., $P_{S_{n}}$, can be expressed as:

(10) $$P_{S_{n}}=\frac{E_{S_{n}}}{\left(1-\alpha \right)T}=\frac{\eta \alpha }{1-\alpha }P_{B}{\left|h_{PS_{n}}\right|}^{2},$$

It needs to be highlighted that (10) gives a general linear power conversion mode on the premise that the wireless device has infinite battery capacity [40].

#### 2.4.2. Saturation Nonlinear Energy Harvesting Mode

In practice, the wireless sensors have limited battery capacity commonly. When the harvested energy exceeds a certain threshold, the output power will reach saturation state [38]. Moreover, the LEH mode is really too simplified, which is not demonstrated by circuit simulations and measurements [11,40]. However, it is quite complicated to deal with the accurate nonlinear mode. Consequently, we hereby adopt a simplified SNEH model, which captures the real characteristics of practical EH circuit closely. It was shown that the SNEH mode is a tractable parameter model [52]. It is worth noting that for ease of analysis, it is assumed that all sensors have the same saturation threshold.

In line with [36,53], for SNEH mode, the output power $P_{S_{n}}$ can be given as:

(11) $$P_{S_{n}}=\left\{\begin{array}{c} \frac{\eta \alpha }{1-\alpha }P_{B}{\left|h_{PS_{n}}\right|}^{2}\;\;\;\;\;if\;\;{\left|h_{PS_{n}}\right|}^{2}<\frac{\Gamma_{th\_S}}{P_{B}}\\ \frac{\eta \alpha }{1-\alpha }\Gamma_{th\_S}\;\;\;\;\;\;\;\;\;\;\;if\;\;{\left|h_{PS_{n}}\right|}^{2}>\frac{\Gamma_{th\_S}}{P_{B}} \end{array}\right.,$$

where $\Gamma_{th\_S}$ denotes the saturation threshold of the receiver at $S_{n}$.

#### 2.4.3. Saturation Nonlinear Energy Harvesting with Activation Threshold

On the other hand, in order to provide continuous power for information transmission, the harvested energy at receiver must be larger than the minimum threshold, which is used to activate the EH circuitry and to sustain the power conversion. Conversely, when the harvested energy is less than the activation threshold, EH circuit keeps inactive resulting in the sensor has not enough power to transmit information. Similarly, it is assumed that all harvesters have the same EH circuit, which means that all sensors have the same saturation threshold and activation threshold. For SNAT mode, the output power $P_{S_{n}}$ can be shown as:

(12) $$P_{S_{n}}=\left\{\begin{array}{c} 0\;\;\;\;\;\;\;\;\;\;\;\;\;\;\;\;\;\;if\;\;{\left|h_{PS_{n}}\right|}^{2}<\frac{\Gamma_{th\_A}}{P_{B}}\;\;\\ \frac{\eta \alpha }{1-\alpha }P_{B}{\left|h_{PS_{n}}\right|}^{2}\;\;\;\;\;if\;\frac{\Gamma_{th\_A}}{P_{B}}<\;{\left|h_{PS_{n}}\right|}^{2}<\frac{\Gamma_{th\_S}}{P_{B}}\\ \frac{\eta \alpha }{1-\alpha }\Gamma_{th\_S}\;\;\;\;\;\;\;\;\;\;\;\;\;if\;\;{\left|h_{PS_{n}}\right|}^{2}>\frac{\Gamma_{th\_S}}{P_{B}} \end{array}\right.,$$

where $\Gamma_{th\_A}$ denotes the activation threshold of the receiver at $S_{n}$.

## 3. Secrecy Performance Analysis

The comprehensive secrecy performance analysis for the multiuser WPSNs with GMS scheme under abovementioned three different EH modes is presented in this section.

### 3.1. Preliminaries

According to [31], the achievable secrecy capacity of the scenario can be given as:

(13) $$C_{s}=\left(1-\alpha \right){\left[\log_{2}\left(1+\gamma_{S_{n^{*}}D}\right)-\log_{2}\left(1+\gamma_{S_{n^{*}}E}\right)\right]}^{+},$$

where $C_{s}$ is the achievable secrecy capacity of the considered multiuser WPSNs, which mainly refers to the difference of the channel capacity between the main channel and the wiretap channel; $\gamma_{S_{n^{*}}D}$ and $\gamma_{S_{n^{*}}E}$ are the instantaneous SNRs at *D* and *E*, respectively, which has been given in Equations (4) and (5); ${\left[x\right]}^{+}=\max\left(x,0\right)$.

It should be noted that for evaluating the secrecy performance, we resort to the SOP as the figure of merit, which is regarded as an important indicator of PLS generally and is mainly used to investigate the secrecy performance of the system when the specific CSI of the wiretap channel cannot be obtained. From information-theoretic sense, when the secrecy capacity $C_{s}$ of the system is lower than a predetermined secrecy rate threshold $R_{th}$, the transmission incurs secrecy outage.

### 3.2. Secrecy Outage Probability Analysis

Specifically, the SOP of each EH mode $P_{sop}^{\left(mod\right)}$ can be shown as

(14) $$P_{sop}^{\left(mod\right)}=\mathrm{Pr}\left\{C_{s}^{\left(mod\right)}<R_{th}\right\}=1-\mathrm{Pr}\left\{C_{s}^{\left(mod\right)}>R_{th}\right\},$$

where $\;\mathrm{mod}\;\in \left\{LEH,SNEH,SNAT\right\}$ and Pr{·} is the probability.

#### 3.2.1. Derivation for LEH Mode

According to Equation (14), the exact SOP of considered multiuser WPSNs with GMS scheme under LEH mode can be formulated as:

(15) $$\begin{array}{c} P_{sop}^{\left(LEH\right)}=1-\sum_{n=1}^{N-K}\left(\begin{array}{c} N\\ K \end{array}\right)\left(\begin{array}{c} N-K\\ n \end{array}\right){\left(-1\right)}^{n}\frac{K\lambda_{SD}{\tilde{\lambda }}_{SE}}{\lambda_{SD}+{\tilde{\lambda }}_{SE}}\;\\ \;\;\;\;\;\;\;\;\;\;\times 2\sqrt{\frac{\xi \lambda_{PS}}{\gamma_{P}\lambda_{SD}\left(n+K\right)}}K_{1}\left(2\sqrt{\frac{\xi \lambda_{PS}\lambda_{SD}\left(n+K\right)}{\gamma_{P}}}\right) \end{array},$$

where $P_{sop}^{\left(LEH\right)}$ is the SOP when the wireless powered sensors operate in the LEH mode, $\gamma_{P}=\frac{P_{B}}{N_{0}}$, $\xi =\frac{\left(1-\alpha \right)\left(\beta -1\right)}{\eta \alpha }$ and $\beta =2^{\frac{R_{th}}{1-\alpha }}$,${\tilde{\lambda }}_{SE}=\frac{\lambda_{SE}}{\beta }$, $K_{1}\left(\cdot \right)$ described in [54] is the modified Bessel function of the second kind.

**Proof.**  Appendix A provides the proof. □

#### 3.2.2. Derivation for SNEH Mode

Similarly, according to (14), the SOP for SNEH is calculated as:

(16) $$\begin{array}{c} P_{sop}^{\left(SNEH\right)}=1-\sum_{n=1}^{N-K}\sum_{k=0}^{+\infty }\left(\begin{array}{c} N\\ K \end{array}\right)\left(\begin{array}{c} N-K\\ n \end{array}\right){\left(-1\right)}^{n+k}\\ \;\;\;\;\;\;\;\;\;\;\;\;\;\times \;\;\frac{K\lambda_{SD}{\tilde{\lambda }}_{SE}}{\lambda_{SD}+{\tilde{\lambda }}_{SE}}\frac{{\left(\frac{\lambda_{PS}\xi }{\gamma_{P}}\right)}^{k}{\left(\frac{\xi }{\Gamma_{S}}\right)}^{-\frac{k}{2}}{\left[\lambda_{SD}\left(n+K\right)\right]}^{\frac{k}{2}-1}}{k!}\\ \;\;\;\;\;\;\;\;\;\;\;\;\times \;e^{-\frac{1}{2}\frac{\xi }{\Gamma_{S}}\lambda_{SD}\left(n+K\right)}W_{-\frac{k}{2},\frac{1-k}{2}}\left[\frac{\xi }{\Gamma_{S}}\lambda_{SD}\left(n+K\right)\right] \end{array},$$

where $P_{sop}^{\left(SNEH\right)}$ is the SOP when the wireless powered sensors work in the SNEH mode, $\Gamma_{S}=\frac{\Gamma_{th\_S}}{N_{0}}$, $W_{\lambda ,\mu }\left(\cdot \right)$ is Whittaker function as defined in [44], Equation (3.381.6).

**Proof.**  Appendix B shows the proof. □

#### 3.2.3. Derivation for SNAT Mode

Furthermore, according to Equation (14), the exact SOP for SNAT is given as:

(17) $$\begin{array}{c} P_{sop}^{\left(SNAT\right)}=1-\sum_{n=1}^{N-K}\sum_{k=0}^{+\infty }\left(\begin{array}{c} N\\ K \end{array}\right)\left(\begin{array}{c} N-K\\ n \end{array}\right){\left(-1\right)}^{n+k}\;\;\;\;\;\;\;\;\\ \;\;\;\;\;\;\;\;\;\;\;\times \;\frac{K\lambda_{SD}{\tilde{\lambda }}_{SE}}{\lambda_{SD}+{\tilde{\lambda }}_{SE}}\frac{{\left(\frac{\lambda_{PS}\xi }{\gamma_{P}}\right)}^{k}{\left(\frac{\xi }{\Gamma_{S}}\right)}^{-\frac{k}{2}}{\left[\lambda_{SD}\left(n+K\right)\right]}^{\frac{k}{2}-1}}{k!}\;\;\;\;\;\;\;\;\;\;\;\;\;\;\;\;\;\;\;\;\\ \;\;\;\;\;\;\;\;\;\;\;\times \;\;e^{-\frac{1}{2}\frac{\xi }{\Gamma_{S}}\lambda_{SD}\left(n+K\right)}W_{-\frac{k}{2},\frac{1-k}{2}}\left[\frac{\xi }{\Gamma_{S}}\lambda_{SD}\left(n+K\right)\right]\\ \;\;\;\;\;\;\;\;\;\;\;+\sum_{n=1}^{N-K}\sum_{k=0}^{+\infty }\left(\begin{array}{c} N\\ K \end{array}\right)\left(\begin{array}{c} N-K\\ n \end{array}\right){\left(-1\right)}^{n+k}\;\;\;\;\;\;\;\;\\ \;\;\;\;\;\;\;\;\;\;\;\times \;\frac{K\lambda_{SD}{\tilde{\lambda }}_{SE}}{\lambda_{SD}+{\tilde{\lambda }}_{SE}}\frac{{\left(\frac{\lambda_{PS}\xi }{\gamma_{P}}\right)}^{k}{\left(\frac{\xi }{\Gamma_{A}}\right)}^{-\frac{k}{2}}{\left[\lambda_{SD}\left(n+K\right)\right]}^{\frac{k}{2}-1}}{k!}\;\;\;\;\;\;\;\;\;\;\;\;\;\;\;\;\;\;\;\;\\ \;\;\;\;\;\;\;\;\;\;\;\times \;\;e^{-\frac{1}{2}\frac{\xi }{\Gamma_{A}}\lambda_{SD}\left(n+K\right)}W_{-\frac{k}{2},\frac{1-k}{2}}\left[\frac{\xi }{\Gamma_{A}}\lambda_{SD}\left(n+K\right)\right]\\ \;\;\;\;\;\;\;\;\;\;\;-\sum_{n=1}^{N-K}\left(\begin{array}{c} N\\ K \end{array}\right)\left(\begin{array}{c} N-K\\ n \end{array}\right)\frac{K\lambda_{SD}{\tilde{\lambda }}_{SE}}{\lambda_{SD}+{\tilde{\lambda }}_{SE}}\;\;\;\;\;\;\;\;\\ \;\;\;\;\;\;\;\;\;\;\;\times \;\frac{1}{\lambda_{SD}\left(n+K\right)}\;\;e^{-\left[\frac{\lambda_{PS}\Gamma_{A}}{\gamma_{B}}+\frac{\xi }{\Gamma_{A}}\lambda_{SD}\left(n+K\right)\right]}\;\;\;\;\;\;\;\;\;\;\;\;\;\;\;\;\;\; \end{array},$$

where $P_{sop}^{\left(SNAT\right)}$ is the SOP when the wireless powered sensors operate in the SNAT mode, $\Gamma_{A}=\frac{\Gamma_{th\_A}}{N_{0}}$.

**Proof.**  Appendix C presents the proof. □

**Remark** **1.** *According to Equations (15)–(17), we find that for the all three EH modes, the number of the source sensors and the EH efficiency factor play a positive role on the SOP of the multiuser WPSNs. On the other hand, the smaller value of the generalized selection coefficient is beneficial for the SOP in three EH modes. Meanwhile, we observe that the secrecy performance of SNEH is better in the case of higher saturation threshold, and the secrecy performance of SNAT is better when the activation threshold is lower. The above discussions will be analyzed and proved specifically in Section 4.*

### 3.3. Secure Energy Efficiency Maximization

In general, the improvement of security often comes at the expense of more energy. In terms of energy-limited WSNs, the excessive pursuit of secrecy improvement has negative impact on the network performance. Therefore, it is valuable to make sure the secure communication with low energy consumption. According to the above analysis, the SEE is used as the proper metric for evaluating the secrecy performance of the multiuser WPSNs, which is commonly adopted to measure the trade-off between the secrecy performance and energy consumption [33]. Generally, the SEE is defined as the ratio between the secure throughput and the total energy consumption. Mathematically, the SEE of above discussed EH modes can be shown as

(18) $$\eta_{s}^{\left(mod\right)}=\frac{R_{th}\left(1-P_{sop}^{\left(mod\right)}\right)}{P_{t}},$$

where $\eta_{s}^{\left(mod\right)}$ denotes the SEE of each EH mode, $P_{t}=\kappa P_{B}+P_{c}$ represents the total power cost at PB, κ denotes the power factor, $P_{c}$ and $P_{B}$ stand for the fixed power and transmit power at PB. It is considered that the harvested energy by the source sensors is fully depleted for information transmission while the power cost of the circuity is neglected.

To find the best transmit power of PB, the SEE maximization problem can be given as

(19) $$\begin{array}{c} \max_{P_{B}}\;\;\;\eta_{s}^{\left(mod\right)}=\frac{R_{th}\left(1-P_{sop}^{\left(mod\right)}\right)}{P_{t}}\\ \;\;s.t.\;\;\;\;\;0<P_{B}\leq P_{\max}, \end{array}$$

where $P_{\max}$ denotes the maximum transmit power of PB. It is obviously that the solving process of the exact expressions for $P_{B}$ is quite complex. Fortunately, with the help of the searching method, we can get the optimal $P_{B}$ based on the simulation and numerical analysis. It is worth noting that the hereinabove SEE optimization problem can be considered to be the guide to the practical implementation, which can be applied to engineering decision-making. Meanwhile, the expression in (19) is more practical for the future communication.

**Remark** **2.** *With the help of Equations (15)–(17), we find that the security performance can be improved by increasing the transmit power in PB. However, the denominator of SEE in (18) is an increasing function of the transmit power. Therefore, overload transmit power has a negative effect on the SEE. On the other hand, increasing time-switching factor can effectively increase the transmit power of the sensors, but also decrease the communication performance of the system due to the reduction of information transmission time. Thus, how to maximize the SEE by optimizing the transmit power in PB and the time-switching factor is of more practical operational significance for the considered multiuser WPSNs.*

## 4. Numerical Results and Discussion

The analytical and simulation results are provided in this section for evaluating the impact of key parameters on the security of the multiuser WPSNs, including the number of source sensors, the EH efficiency factor, the generalized selection coefficient, the saturation threshold, the activation threshold, the transmit power of PB and the time-switching factor. It is worth noting that all the numerical results in this section come from the simulation environment of MATLAB, which all are true experimental results. Combining the parameter setting in the article with the program code, all the results can be reproduced. Unless otherwise stated, the system parameters and the initial conditions are set as below: N=3, K=2, η=0.6, ($R_{th}$ = 0.1 bits/s/Hz), $\gamma_{P}=30\;\mathrm{dB}$, α=0.5, $\Gamma_{th\_S}=10\;\mathrm{dB}$, $\Gamma_{th\_A}=0\;\mathrm{dB}$, k=0.01, $P_{c}=100\;\mathrm{mW}$ and $\lambda_{PS}=\lambda_{SD}=1$. Specifically, the times of the simulation set to 100000. It should be emphasized that the ratio of the main channel to the eavesdropping channel can be expressed as $\tau =\frac{\lambda_{SE}}{\lambda_{SD}}$ [36], whose value is considered between −10dB and 50dB in this section. From the figures, it is obvious that the simulation results are in accurate agreement with the theoretical calculation results, which verifies the correctness of our derivations.

**Figure 3:**
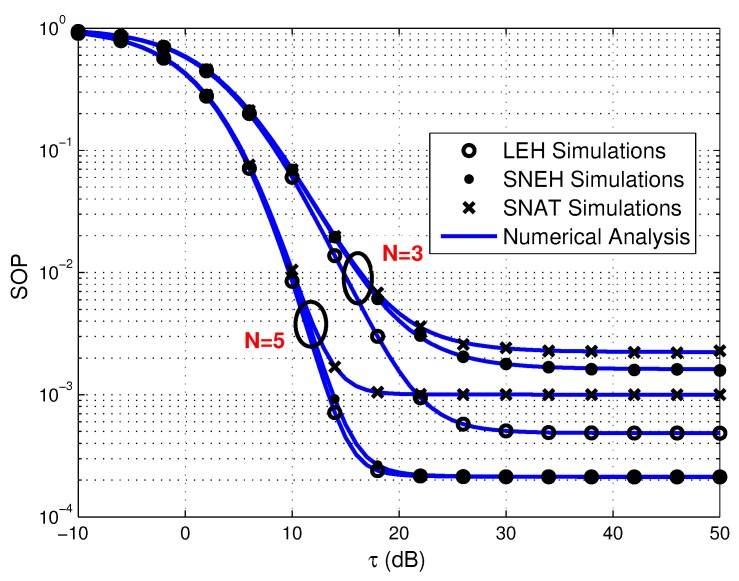
Secrecy outage probability versus τ for three EH modes under various *N*, where $N=\left\{3,5\right\}$.

**Figure 4:**
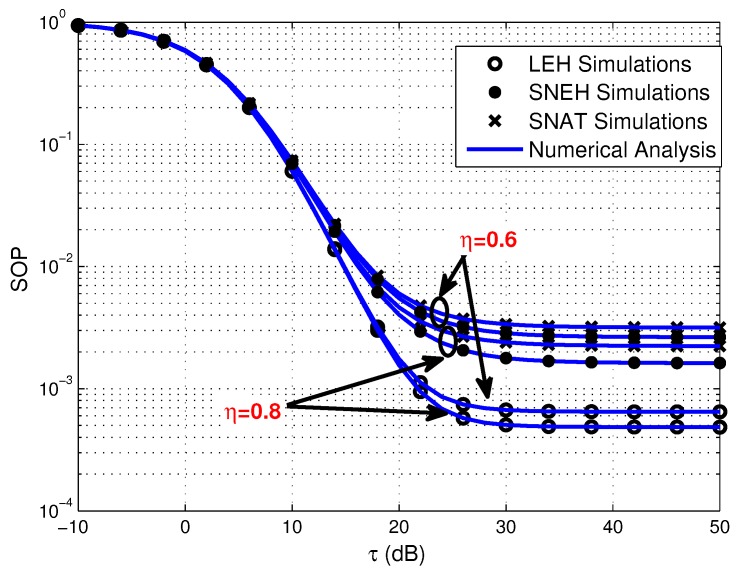
Secrecy outage probability versus τ for three EH modes under various η, where $\eta =\left\{0.6,0.8\right\}$.

**Figure 5:**
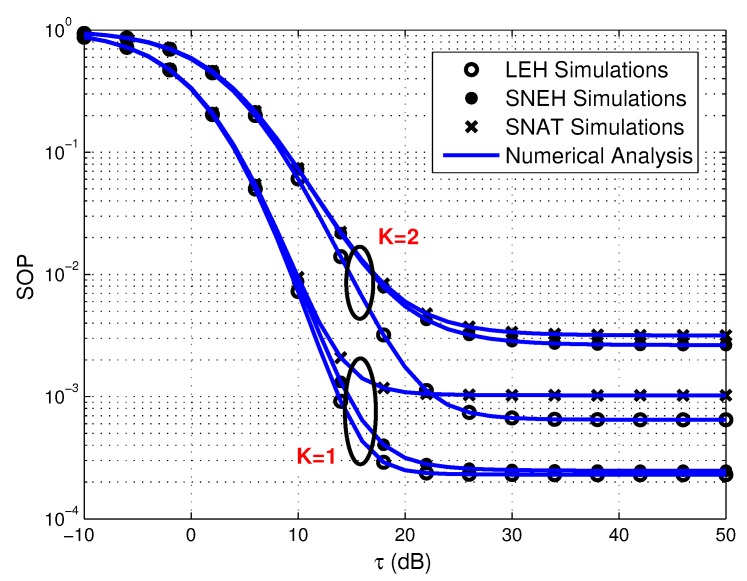
Secrecy outage probability versus τ for three EH modes under various *K*, where $K=\left\{1,2\right\}$.

As shown in Figure 3, Figure 4 and Figure 5, we provide the SOP of the considered multiuser WPSNs with GMS versus τ for three different EH modes under various *N*, η, and *K*. From Figure 3, we can observe that for the all three EH modes, the number of the source sensors has a positive impact on the SOP, i.e., a lager value of *N* brings about better SOP performance. The reason is that the better sensor scheduling advantage can be acquired when more alternative sensors are available, which can provide better multiuser diversity gain. Figure 4 illustrates that the EH efficiency factor plays a positive role on the SOP. This is due to the fact that a higher energy conversion efficiency contributes to harvest more energy and power for secure communication. On the other hand, Figure 5 shows that the smaller value of *K* is beneficial for the SOP in three EH modes. It can be explained that a better sensor in terms of secrecy capacity is chosen, which is helpful to enhance the security of the system.

**Figure 6:**
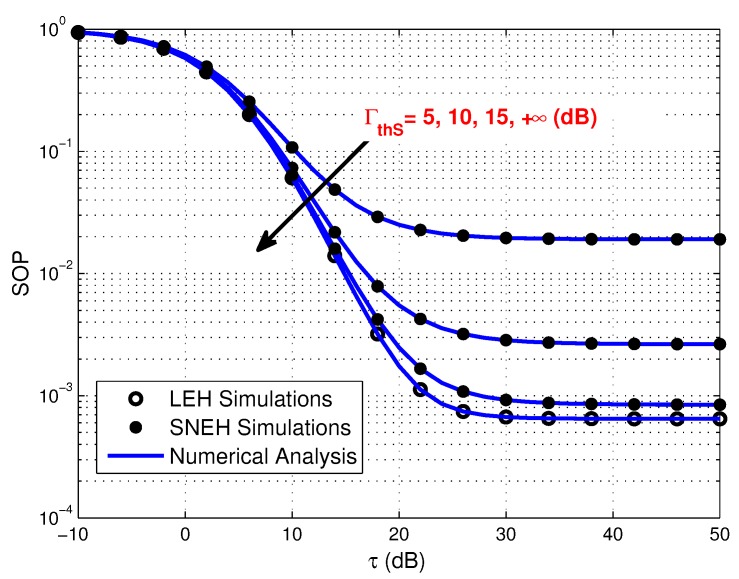
Secrecy outage probability versus τ for LEH and SNEH under various $\Gamma_{th\_S}$, where $\Gamma_{th\_S}=\left\{5,10,15,+\infty \right\}$.

Figure 6 depicts the SOP versus τ for LEH and SNEH under different saturation thresholds. It is shown that the secrecy performance of SNEH is better in the case of the saturation threshold $\Gamma_{th\_S}$ is higher on account of that batteries with large capability can provide more stored energy for secure communication. However, it exists a floor, in which the SOP keeps unchanged as $\Gamma_{th\_S}$ increases. This is because increasing $\Gamma_{th\_S}$ can only enhance the battery capability rather than the EH capacity. In addition, it is noted that LEH is an ideal situation for SNEH when the saturation threshold is large enough.

**Figure 7:**
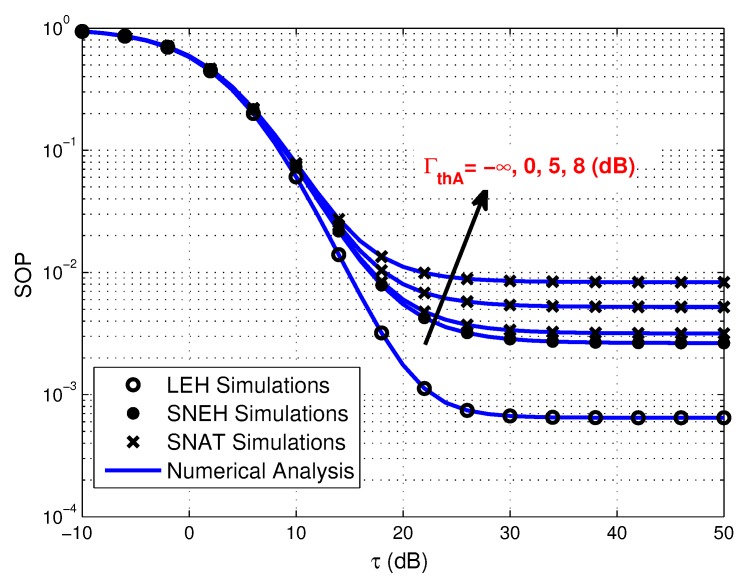
Secrecy outage probability versus τ for three EH modes under various $\Gamma_{th\_A}$, where $\Gamma_{th\_S}=10$ dB and $\Gamma_{th\_A}=\left\{-\infty ,0,5,8\right\}$.

Figure 7 presents the SOP versus τ in three different EH modes under different activation threshold. As can be easily observed that the secrecy performance of SNAT is better when the activation threshold is lower. This can be explained by the fact that the lower activation threshold is beneficial for more source users to participate in the information transmission, which makes the WPSNs to get better multiuser diversity gain. On the other hand, as shown in the figure, it can be inferred that SNEH is a special situation for SNAT when the activation threshold is lower enough.

**Figure 8:**
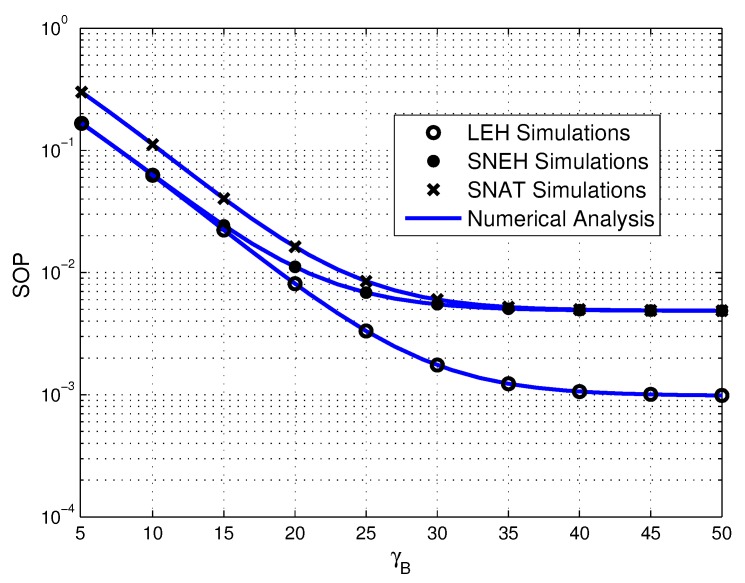
Secrecy outage probability versus $\gamma_{P}$ under three EH modes, where τ=20 dB.

Figure 8 plots the impact of the transmit power of PB on SOP for three different EH modes. As shown clearly in the figure that the SOP of LEH and SNEH is almost identical when the transmit power of PB is lower. The reason is that the lower transmit power of PB makes the energy acquired in all sensors difficult to achieve the saturation threshold, leading to the multiuser WPSN operates in the LEH mode. In contrast, the SOP of SNAT and SNEH is almost consistent when the transmit power of PB is higher. It is because the large transmit power of PB contributes to activate all the source sensors to harvest energy for information transmission, which is leading to all the sensors of SNAT work in the SNEH mode.

**Figure 9:**
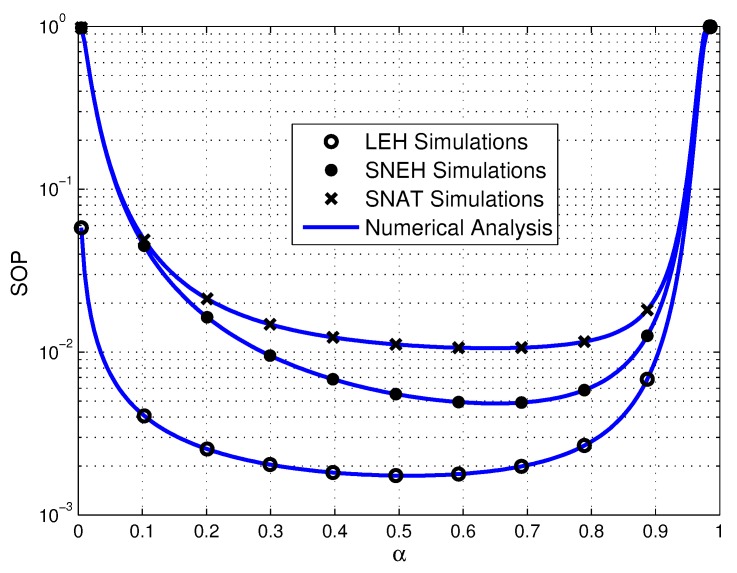
Secrecy outage probability versus α in three EH modes, where τ=20 dB.

Figure 9 shows the SOP performance under various α in three different EH modes. It can be observed that the function of SOP and α are both unimodal function. The reason is that for the smaller value of α, which means that the time for EH is less, the harvested energy is usually insufficient for the operation of multiple source sensors. On the contrary, when α is large enough, the time for information transmission will be limited seriously, which in turn leads to high communication interruption probability. Consequently, α should be considered carefully to provide efficient communication.

**Figure 10:**
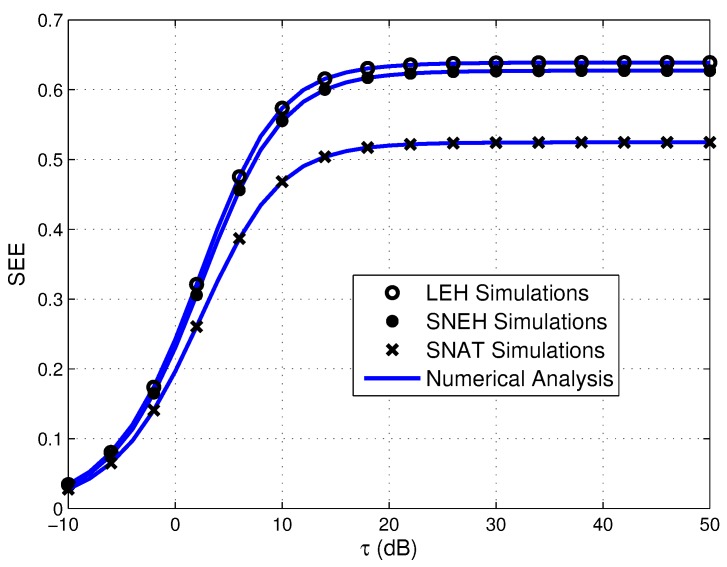
Secure energy efficiency versus τ in three EH modes, where $\Gamma_{th\_S}=3$ dB.

Figure 10 describes the SEE of the considered WPSN versus τ in different EH modes. From the figure, we can find that the activation threshold limits the secrecy performance of the system in SNAT mode. By comparison, the impact of the saturation threshold on the security in SNEH mode is relatively small. Therefore, it is very valuable to investigate the influence of activation threshold on secrecy performance for WPSNs.

## 5. Conclusions

In this paper, we explored the PLS in the multiuser WPSNs with GMS scheme. First, we analyzed the impact of various key parameters on the secrecy performance of the system, including the number of source sensor, the EH efficiency factor, the generalized selection coefficient, the saturation threshold, the activation threshold, the transmit power of PB and the time-switching coefficient. Furthermore, to get a deeper insight, we discussed the maximization problem of SEE and compared the SEE under three different EH modes. Simulation results demonstrated that: (1) the number of source sensors, the EH efficiency and the transmit power of PB all have positive impact on SOP, the smaller generalized selection coefficient is advantageous for secrecy performance; (2) LEH is an ideal situation for SNEH when the saturation threshold is large enough and SNEH is a special situation for SNAT when the activation threshold is lower enough; (3) the time-switching factor and the activation threshold have an important effect on the secrecy performance, which should be considered carefully. It is worth noting that the more complex topology, such as the network following the random geometric distribution, is not considered in this paper, which can be explored in our future works. In addition, the model of the EH nonlinearity in this paper is relatively simple and basic, only the characteristics of the rectifier are preliminarily described, and the next step is to consider a more practical rectifier model. 

## Appendix A

First, from Equation (14), $P_{sop}^{\left(LEH\right)}$ can be given as

(A1) $$P_{sop}^{\left(LEH\right)}=1-\mathrm{Pr}\left\{C_{s}^{\left(LEH\right)}>R_{th}\right\}\;$$

Furthermore, with the help of (4), (5), (10) and (13), $\mathrm{Pr}\left\{C_{s}^{\left(LEH\right)}>R_{th}\right\}\;$ can be derived and the expansion is listed below:

(A2) $$\begin{array}{c} \mathrm{Pr}\left\{C_{s}^{\left(LEH\right)}>R_{th}\right\}=\;\mathrm{Pr}\left\{\frac{1+\gamma_{S_{n^{*}}D}}{1+\gamma_{S_{n^{*}}E}}>2^{\frac{R_{th}}{1-\alpha }}\right\}\;\;\;\\ =\;\;\mathrm{Pr}\left\{\gamma_{S_{n^{*}}D}>\beta \gamma_{S_{n^{*}}E}+\left(\beta -1\right)\right\}\;\;\;\\ =\;\;\mathrm{Pr}\left\{\frac{\eta \alpha }{1-\alpha }{\left|h_{PS_{n^{*}}}\right|}^{2}\gamma_{B}\left({\left|h_{S_{n^{*}}D}\right|}^{2}-\beta {\left|h_{S_{n^{*}}E}\right|}^{2}\right)>\beta -1\right\}\\ =\;\;\mathrm{Pr}\left\{{\left|h_{PS_{n^{*}}}\right|}^{2}>\frac{\xi }{Z\gamma_{P}}\right\}\;\;\; \end{array}$$

where $Z={\left|h_{S_{n^{*}}D}\right|}^{2}-\beta {\left|h_{S_{n^{*}}E}\right|}^{2}$.

By introducing the result in [28], Equation (39) and aided by Equations (8) and (9), the PDF of *Z* can be further expressed as

(A3) $$\begin{array}{c} f_{Z}\left(z\right)=\int_{0}^{+\infty }f_{{\left|h_{S_{n^{*}}D}\right|}^{2}}\left(z+y\right)\frac{1}{\beta }f_{{\left|h_{S_{n^{*}}E}\right|}^{2}}\left(\frac{y}{\beta }\right)dy\\ \;\;\;\;\;\;\;\;\;\;=\sum_{n=1}^{N-K}\left(\begin{array}{c} N\\ K \end{array}\right)\left(\begin{array}{c} N-K\\ n \end{array}\right){\left(-1\right)}^{n}\frac{K\lambda_{SD}{\tilde{\lambda }}_{SE}}{\lambda_{SD}+{\tilde{\lambda }}_{SE}}\\ \;\;\;\;\;\;\;\;\;\;\times \exp\left[-\lambda_{SD}\left(n+K\right)z\right] \end{array}$$

Then, by substituting Equation (A3) into Equation (A2) and with the help of [44], Equation (3.324.1), we have

(A4) $$\begin{array}{c} \mathrm{Pr}\left\{C_{s}^{\left(LEH\right)}>R_{th}\right\}=\int_{0}^{+\infty }\int_{\frac{\xi }{Z\gamma_{P}}}^{+\infty }f_{{\left|h_{PS_{n^{*}}}\right|}^{2}}\left(x\right)f_{Z}\left(z\right)dxdz\\ \;\;\;\;\;\;\;\;\;\;\;\;\;\;\;\;=\sum_{n=1}^{N-K}\left(\begin{array}{c} N\\ K \end{array}\right)\left(\begin{array}{c} N-K\\ n \end{array}\right){\left(-1\right)}^{n}\frac{K\lambda_{SD}{\tilde{\lambda }}_{SE}}{\lambda_{SD}+{\tilde{\lambda }}_{SE}}\\ \;\;\;\;\;\;\;\;\;\;\;\;\;\;\;\;\times \int_{0}^{+\infty }\exp\left[-\frac{\frac{4\lambda_{PS}\xi }{\gamma_{P}}}{4z}-\lambda_{SD}\left(n+K\right)z\right]dz\\ \;\;\;\;\;\;\;\;\;\;\;\;\;\;\;\;=\sum_{n=1}^{N-K}\left(\begin{array}{c} N\\ K \end{array}\right)\left(\begin{array}{c} N-K\\ n \end{array}\right){\left(-1\right)}^{n}\frac{K\lambda_{SD}{\tilde{\lambda }}_{SE}}{\lambda_{SD}+{\tilde{\lambda }}_{SE}}\\ \;\;\;\;\;\;\;\;\;\;\;\;\;\;\;\;\times 2\sqrt{\frac{\xi \lambda_{PS}}{\gamma_{P}\lambda_{SD}\left(n+K\right)}}K_{1}\left(2\sqrt{\frac{\xi \lambda_{PS}\lambda_{SD}\left(n+K\right)}{\gamma_{P}}}\right) \end{array}$$

Finally, by substitutinglEqution (A4) into Eqution(A1), $P_{sop}^{\left(LEH\right)}$ in Eqution (15) can be derived.

## Appendix B

Following the same line of derivation used for obtaining $P_{sop}^{\left(LEH\right)}$, from Eqution (14), $P_{sop}^{\left(SNEH\right)}$ can be shown as

(A5) $$P_{sop}^{\left(SNEH\right)}=1-\mathrm{Pr}\left\{C_{s}^{\left(SNEH\right)}>R_{th}\right\}\;$$

With the help of Equtions (4), (5), (11) and (13), $\mathrm{Pr}\left\{C_{s}^{\left(SNEH\right)}>R_{th}\right\}\;$ can be obtained and the expansion is listed below:

(A6) $$\begin{array}{c} \mathrm{Pr}\left\{C_{s}^{\left(SNEH\right)}>R_{th}\right\}=\;\;\mathrm{Pr}\left\{\gamma_{S_{n^{*}}D}>\beta \gamma_{S_{n^{*}}E}+\left(\beta -1\right)\right\}\\ =\mathrm{Pr}\left\{\begin{array}{c} \frac{\eta \alpha }{1-\alpha }{\left|h_{PS_{n^{*}}}\right|}^{2}\gamma_{B}\left({\left|h_{S_{n^{*}}D}\right|}^{2}-\beta {\left|h_{S_{n^{*}}E}\right|}^{2}\right)>\beta -1\;,\;\\ {\left|h_{PS_{n^{*}}}\right|}^{2}<\frac{\Gamma_{th\_S}}{P_{B}} \end{array}\right\}\\ +\mathrm{Pr}\left\{\begin{array}{c} \frac{\eta \alpha }{1-\alpha }\frac{\Gamma_{th\_S}}{N_{0}}\left({\left|h_{S_{n^{*}}D}\right|}^{2}-\beta {\left|h_{S_{n^{*}}E}\right|}^{2}\right)>\beta -1\;,\\ \;{\left|h_{PS_{n^{*}}}\right|}^{2}>\frac{\Gamma_{th\_S}}{P_{B}} \end{array}\right\}\\ =\mathrm{Pr}\left\{\;{\left|h_{PS_{n^{*}}}\right|}^{2}>\frac{\xi }{\gamma_{P}Z}\;,\;{\left|h_{PS_{n^{*}}}\right|}^{2}<\frac{\Gamma_{S}}{\gamma_{P}}\right\}\\ +\mathrm{Pr}\left\{Z>\frac{\xi }{\Gamma_{S}}\;,\;\;{\left|h_{PS_{n^{*}}}\right|}^{2}>\frac{\Gamma_{S}}{\gamma_{P}}\right\}\\ =\mathrm{Pr}\left\{\;\frac{\xi }{\gamma_{P}Z}<\;{\left|h_{PS_{n^{*}}}\right|}^{2}<\frac{\Gamma_{S}}{\gamma_{P}}\right\}\\ +\mathrm{Pr}\left\{Z>\frac{\xi }{\Gamma_{S}}\;\right\}\mathrm{Pr}\left\{\;{\left|h_{PS_{n^{*}}}\right|}^{2}>\frac{\Gamma_{S}}{\gamma_{P}}\right\} \end{array}$$

Then, by substituting Eqution (A3) into Eqution(A6) and using the Maclaurin series of the term $e^{-\frac{\mu }{x}}=\sum_{k=0}^{\infty }\frac{{\left(-1\right)}^{k}\mu^{k}}{k!x^{k}}$ [55], aided by the [44], Equation (3.381.6), i.e., Whittaker function, we have

(A7) $$\begin{array}{c} \mathrm{Pr}\left\{C_{s}^{\left(SNEH\right)}>R_{th}\right\}=\int_{\frac{\xi }{\Gamma_{S}}}^{+\infty }\exp\left\{-\frac{\xi }{\gamma_{P}z}\lambda_{PS}\right\}f_{Z}\left(z\right)dz\\ \;\;\;\;\;\;\;\;\;\;\;\;\;\;\;\;=\sum_{n=1}^{N-K}\left(\begin{array}{c} N\\ K \end{array}\right)\left(\begin{array}{c} N-K\\ n \end{array}\right){\left(-1\right)}^{n}\frac{K\lambda_{SD}{\tilde{\lambda }}_{SE}}{\lambda_{SD}+{\tilde{\lambda }}_{SE}}\\ \;\;\;\;\;\;\;\;\;\;\;\;\;\;\;\;\times \int_{\frac{\xi }{\Gamma_{S}}}^{+\infty }\exp\left[-\frac{\frac{\lambda_{PS}\xi }{\gamma_{P}}}{z}-\lambda_{SD}\left(n+K\right)z\right]dz\\ \;\;\;\;\;\;\;\;\;\;\;\;\;\;\;\;=\sum_{n=1}^{N-K}\sum_{k=0}^{+\infty }\left(\begin{array}{c} N\\ K \end{array}\right)\left(\begin{array}{c} N-K\\ n \end{array}\right){\left(-1\right)}^{n+k}\\ \;\;\;\;\;\;\;\;\;\;\;\;\;\;\;\;\times \frac{K\lambda_{SD}{\tilde{\lambda }}_{SE}}{\lambda_{SD}+{\tilde{\lambda }}_{SE}}\frac{{\left(\frac{\lambda_{PS}\xi }{\gamma_{P}}\right)}^{k}{\left(\frac{\xi }{\Gamma_{S}}\right)}^{-\frac{k}{2}}{\left[\lambda_{SD}\left(n+K\right)\right]}^{\frac{k}{2}-1}}{k!}\\ \;\;\;\;\;\;\;\;\;\;\;\;\;\;\;\;\times \;\;e^{-\frac{1}{2}\frac{\xi }{\Gamma_{S}}\lambda_{SD}\left(n+K\right)}W_{-\frac{k}{2},\frac{1-k}{2}}\left[\frac{\xi }{\Gamma_{S}}\lambda_{SD}\left(n+K\right)\right] \end{array}$$

Finally, by substituting Eqution (A7) into Eqution (A5), $P_{sop}^{\left(SNEH\right)}$ in Eqution (16) can be obtained.

## Appendix C

Similarly, from Eqution (14), we can derive $P_{sop}^{\left(SNAT\right)}$ as

(A8) $$P_{sop}^{\left(SNAT\right)}=1-\mathrm{Pr}\left\{C_{s}^{\left(SNAT\right)}>R_{th}\right\}\;$$

With the help of Equtions (4), (5), (12) and (13), $\mathrm{Pr}\left\{C_{s}^{\left(SNAT\right)}>R_{th}\right\}\;$ can be obtained and the expansion is listed below as Eqution (A9):

(A9) $$\begin{array}{c} \mathrm{Pr}\left\{C_{s}^{\left(SNAT\right)}>R_{th}\right\}=\;\;\mathrm{Pr}\left\{\gamma_{S_{n^{*}}D}>\beta \gamma_{S_{n^{*}}E}+\left(\beta -1\right)\right\}\\ =\mathrm{Pr}\left\{\begin{array}{c} \frac{\eta \alpha }{1-\alpha }{\left|h_{PS_{n^{*}}}\right|}^{2}\gamma_{B}\left({\left|h_{S_{n^{*}}D}\right|}^{2}-\beta {\left|h_{S_{n^{*}}E}\right|}^{2}\right)>\beta -1\;,\;\\ \frac{\Gamma_{th\_A}}{P_{B}}<{\left|h_{PS_{n^{*}}}\right|}^{2}<\frac{\Gamma_{th\_S}}{P_{B}} \end{array}\right\}\\ +\mathrm{Pr}\left\{\begin{array}{c} \frac{\eta \alpha }{1-\alpha }\frac{\Gamma_{th\_S}}{N_{0}}\left({\left|h_{S_{n^{*}}D}\right|}^{2}-\beta {\left|h_{S_{n^{*}}E}\right|}^{2}\right)>\beta -1\;,\\ \;{\left|h_{PS_{n^{*}}}\right|}^{2}>\frac{\Gamma_{th\_S}}{P_{B}} \end{array}\right\}\\ =\mathrm{Pr}\left\{\;{\left|h_{PS_{n^{*}}}\right|}^{2}>\frac{\xi }{\gamma_{P}Z}\;,\;\frac{\Gamma_{A}}{\gamma_{P}}<{\left|h_{PS_{n^{*}}}\right|}^{2}<\frac{\Gamma_{S}}{\gamma_{P}}\right\}\\ +\mathrm{Pr}\left\{Z>\frac{\xi }{\Gamma_{S}}\;,\;\;{\left|h_{PS_{n^{*}}}\right|}^{2}>\frac{\Gamma_{S}}{\gamma_{P}}\right\}\\ =\mathrm{Pr}\left\{\;{\left|h_{PS_{n^{*}}}\right|}^{2}>\frac{\xi }{\gamma_{P}Z}\;,\;\frac{\Gamma_{A}}{\gamma_{P}}<{\left|h_{PS_{n^{*}}}\right|}^{2}<\frac{\Gamma_{S}}{\gamma_{P}}\right\}\\ +\mathrm{Pr}\left\{Z>\frac{\xi }{\Gamma_{S}}\;\right\}\mathrm{Pr}\left\{\;{\left|h_{PS_{n^{*}}}\right|}^{2}>\frac{\Gamma_{S}}{\gamma_{P}}\right\} \end{array}$$

Then, by substituting Eqution (A3) into Equation (A9) and using the Maclaurin series of the term $e^{-\frac{\mu }{x}}=\sum_{k=0}^{\infty }\frac{{\left(-1\right)}^{k}\mu^{k}}{k!x^{k}}$ [55], with the help of [44], Equation (3.381.6), we have

(A10) $$\begin{array}{c} \mathrm{Pr}\left\{C_{s}^{\left(SNAT\right)}>R_{th}\right\}\\ \;\;\;\;\;\;\;\;\;\;\;\;\;\;\;\;\;\;\;=\;\int_{\frac{\xi }{\Gamma_{A}}}^{+\infty }\mathrm{Pr}\left\{\frac{\Gamma_{A}}{\gamma_{P}}<{\left|h_{PS_{n^{*}}}\right|}^{2}<\frac{\Gamma_{S}}{\gamma_{P}}\right\}f_{Z}\left(z\right)dz\\ \;\;\;\;\;\;\;\;\;\;\;\;\;\;\;\;\;\;\;+\int_{\frac{\xi }{\Gamma_{S}}}^{\frac{\xi }{\Gamma_{A}}}\mathrm{Pr}\left\{\frac{\xi }{\gamma_{P}z}<{\left|h_{PS_{n^{*}}}\right|}^{2}<\frac{\Gamma_{S}}{\gamma_{P}}\right\}f_{Z}\left(z\right)dz\\ \;\;\;\;\;\;\;\;\;\;\;\;\;\;\;\;\;\;\;+\int_{\frac{\xi }{\Gamma_{S}}}^{+\infty }\mathrm{Pr}\left\{{\left|h_{PS_{n^{*}}}\right|}^{2}>\frac{\Gamma_{S}}{\gamma_{P}}\right\}f_{Z}\left(z\right)dz\\ \;\;\;\;\;\;\;\;\;\;\;\;\;\;\;\;\;\;\;=\int_{\frac{\xi }{\Gamma_{S}}}^{+\infty }\mathrm{Pr}\left\{{\left|h_{PS_{n^{*}}}\right|}^{2}>\frac{\xi }{\gamma_{P}z}\right\}f_{Z}\left(z\right)dz\\ \;\;\;\;\;\;\;\;\;\;\;\;\;\;\;\;\;\;\;-\int_{\frac{\xi }{\Gamma_{A}}}^{+\infty }\mathrm{Pr}\left\{{\left|h_{PS_{n^{*}}}\right|}^{2}>\frac{\xi }{\gamma_{P}z}\right\}f_{Z}\left(z\right)dz\\ \;\;\;\;\;\;\;\;\;\;\;\;\;\;\;\;\;\;\;+\int_{\frac{\xi }{\Gamma_{A}}}^{+\infty }\mathrm{Pr}\left\{{\left|h_{PS_{n^{*}}}\right|}^{2}>\frac{\Gamma_{A}}{\gamma_{P}}\right\}f_{Z}\left(z\right)dz\\ \;\;\;\;\;\;\;\;\;\;\;\;\;\;\;\;=\sum_{n=1}^{N-K}\sum_{k=0}^{+\infty }\left(\begin{array}{c} N\\ K \end{array}\right)\left(\begin{array}{c} N-K\\ n \end{array}\right){\left(-1\right)}^{n+k}\\ \;\;\;\;\;\;\;\;\;\;\;\;\;\;\;\;\times \frac{K\lambda_{SD}{\tilde{\lambda }}_{SE}}{\lambda_{SD}+{\tilde{\lambda }}_{SE}}\frac{{\left(\frac{\lambda_{PS}\xi }{\gamma_{P}}\right)}^{k}{\left(\frac{\xi }{\Gamma_{S}}\right)}^{-\frac{k}{2}}{\left[\lambda_{SD}\left(n+K\right)\right]}^{\frac{k}{2}-1}}{k!}\\ \;\;\;\;\;\;\;\;\;\;\;\;\;\;\;\;\times \;\;e^{-\frac{1}{2}\frac{\xi }{\Gamma_{S}}\lambda_{SD}\left(n+K\right)}W_{-\frac{k}{2},\frac{1-k}{2}}\left[\frac{\xi }{\Gamma_{S}}\lambda_{SD}\left(n+K\right)\right]\\ \;\;\;\;\;\;\;\;\;\;\;\;\;\;\;\;-\sum_{n=1}^{N-K}\sum_{k=0}^{+\infty }\left(\begin{array}{c} N\\ K \end{array}\right)\left(\begin{array}{c} N-K\\ n \end{array}\right){\left(-1\right)}^{n+k}\\ \;\;\;\;\;\;\;\;\;\;\;\;\;\;\;\;\times \frac{K\lambda_{SD}{\tilde{\lambda }}_{SE}}{\lambda_{SD}+{\tilde{\lambda }}_{SE}}\frac{{\left(\frac{\lambda_{PS}\xi }{\gamma_{P}}\right)}^{k}{\left(\frac{\xi }{\Gamma_{A}}\right)}^{-\frac{k}{2}}{\left[\lambda_{SD}\left(n+K\right)\right]}^{\frac{k}{2}-1}}{k!}\\ \;\;\;\;\;\;\;\;\;\;\;\;\;\;\;\;\times \;\;e^{-\frac{1}{2}\frac{\xi }{\Gamma_{A}}\lambda_{SD}\left(n+K\right)}W_{-\frac{k}{2},\frac{1-k}{2}}\left[\frac{\xi }{\Gamma_{A}}\lambda_{SD}\left(n+K\right)\right]\\ \;\;\;\;\;\;\;\;\;\;\;\;\;\;\;\;+\sum_{n=1}^{N-K}\left(\begin{array}{c} N\\ K \end{array}\right)\left(\begin{array}{c} N-K\\ n \end{array}\right){\left(-1\right)}^{n}\frac{K\lambda_{SD}{\tilde{\lambda }}_{SE}}{\lambda_{SD}+{\tilde{\lambda }}_{SE}}\\ \;\;\;\;\;\;\;\;\;\;\;\;\;\;\;\;\;\times \frac{1}{\lambda_{SD}\left(n+K\right)}e^{-\left[\frac{\lambda_{PS}\Gamma_{A}}{\gamma_{P}}+\frac{\xi }{\Gamma_{A}}\lambda_{SD}\left(n+K\right)\right]} \end{array}$$

Finally, by substituting Eqution (A10) into Eqution(A8), we have $P_{sop}^{\left(SNEH\right)}$ in Eqution (17).

## References

[1] Akpakwu G.A., Silva B.J., Hancke G.P., Abu-Mahfouz A.M. (2018). A survey on 5G networks for the Internet of Things: Communication technologies and challenges. IEEE Access.

[2] Agiwal M., Roy A., Saxena N. (2016). Next generation 5G wireless networks: A comprehensive survey. IEEE Commun. Surv. Tutor..

[3] Atzori L., Iera A., Morabito G. (2010). The internet of things: A survey. Comput. Netw..

[4] Li Q., Feng G., Leung V. (2016). Optimal Transmission Policies for Relay Communication Networks with Ambient Energy Harvesting Relays. IEEE J. Sel. Areas Commun..

[5] Sudevalayam S., Kulkarni P. (2011). Energy Harvesting Sensor Nodes: Survey and Implications. IEEE Commun. Surv. Tutor..

[6] Rajesh R., Sharma V., Viswanath P. (2014). Capacity of guassian channels with energy harvesting and processing cost. IEEE Trans. Inf. Theory.

[7] Alevizos P.N., Bletsas A. (2018). Sensitive and nonlinear far-field RF energy harvesting in wireless communications. IEEE Trans. Wirel. Commun..

[8] Bi S., Ho C.K., Zhang R. (2015). Wireless powered communication: Opportunities and challenges. IEEE Commun. Mag..

[9] Bi S., Zeng Y.G., Zhang R. (2016). Wireless powered communication networks: An overview. IEEE Trans. Wirel. Commun..

[10] Tabassum H., Hossain E., Ogundipe A., Kim D.I. (2015). Wireless powered cellular networks: Key challenges and solution techniques. IEEE Commun. Mag..

[11] Niyato D., Kim D.I., Miso M., Han Z. (2017). Wireless powered comunication networks: Research direction and technological approaches. IEEE Wirel. Commun..

[12] Ramezani P., Jamalipour A. (2017). Toward the evolution of wireless powered communication networks for the future internet of things. IEEE Netw..

[13] Chen H., Zhai C., Li Y., Vucetic B. (2018). Cooperative strategies for wireless-powered communications: An overview. IEEE Wirel. Commun..

[14] Ortega Y.R., Upadhyay P.K., da Costa D.B., Bithas P.S., Kanatas A.G., Dias U.S., de Sousa Junior R.T. (2017). Joint effect of jamming and noise on the secrecy outage performance of wiretap channels with feedback delay and multiple antennas. IEEE Trans. Emerg. Telecommun. Technol..

[15] Bloch M., Barros J. (2011). Physical-Layer Security: From Information Theory to Security Engineering.

[16] Rodriguez L.J., Tran N.H., Duong T.Q., Le-Ngoc T., Elkashlan M., Shetty S. (2015). Physical layer security in wireless cooperative relay networks: State of the art and beyond. IEEE Commun. Mag..

[17] Hamamreh J.M., Furqan H.M., Arslan H. (2019). Classifications and applications of physical layer security techniques for confidentiality: A comprehensive survey. IEEE Commun. Surv. Tutor..

[18] Lei H., Dai Z., Park K., Lei W., Pan G., Alouini M. (2018). Secrecy outage analysis of mixed RF-FSO downlink SWIPT systems. IEEE Trans. Commun..

[19] Lei H., Xu M., Ansari I.S., Pan G., Qaraqe K.A., Alouini M. (2017). On secure underlay MIMO cognitive radio networks with energy harvesting and transmit antenna selection. IEEE Trans. Green Commun. Netw..

[20] Lee K., Lim J.-T., Choi H.-H. (2019). Impact of Outdated CSI on the Secrecy Performance of Wireless-Powered Untrusted Relay Networks. IEEE Trans. Inf. Forensics Secur..

[21] Sun X., Yang W., Cai Y., Xiang Z., Tang X. (2019). Secure transmissions in millimeter wave SWIPT UAV-based relay networks. IEEE Wirel. Commun Lett..

[22] Ng D., Lo E., Schober R. (2014). Robust beamforming for secure communication in systems with wireless information and power transfer. IEEE Trans. Wirel. Commun..

[23] Pan G., Tang C., Li T., Chen Y. (2015). Secrecy performance analysis for SIMO simultaneous wireless information and power transfer systems. IEEE Trans. Commun..

[24] Shi Q., Xu W., Wu J., Song E., Wang Y. (2015). Secure beamforming for MIMO broadcast with wireless information and power transfer. IEEE Trans. Wirel. Commun..

[25] Liu W., Zhou X., Durrani S., Popovski P. (2016). Secure communication with a wireless powered friendly jammer. IEEE Trans. Wirel. Commun..

[26] Moon J., Lee H., Song C., Lee I. (2017). Secrecy performance optimization for wireless powered communication networks with an energy harvesting jammer. IEEE Trans. Commun..

[27] Shafie A.E., Niyato D., Al-Dhahir N. (2017). Security of an ordered-based distributive jamming scheme. IEEE Wirel. Commun Lett..

[28] Li B., Fei Z., Chen H. (2016). Robust artificial noise-aided secure beamforming in wireless powered non-regenerative relay networks. IEEE Access.

[29] Zhao M., Wang X., Feng S. (2015). Joint power splitting and secure beamforming design in the multiple non-regenerative wireless-powered relay networks. IEEE Commun. Lett..

[30] Xing H., Wong K.K., Nalllanathan A., Zhang R. (2016). Wireless powered cooperative jamming for secrecy multi-AF relaying networks. IEEE Trans. Wirel. Commun..

[31] Nguyen N.P., Duong T.Q., Ngo H.Q., Hadzi-Velkov Z., Shu L. (2016). Secure 5G wireless communications: A joint relay selection and wireless power transfer approach. IEEE Access.

[32] Vo V.N., Nguyen T.G., So-In C., Baig Z.A., Sanguanpong S. (2018). Secrecy outage performance analysis for energy harvesting sensor networks with a jammer using relay selection strategy. IEEE Access.

[33] Wang Y., Yang W., Shang X., Hu J., Huang Y., Cai Y. (2018). Energy-efficient secure transmission for wireless powered internet of things with multiple power beacons. IEEE Access.

[34] Yang M., Guo D., Huang Y., Duong T.Q., Zhang B. (2016). Secure multiuser scheduling in downlink dual-hop regenerative relay networks over Nakagami-m fading channels. IEEE Trans. Wirel. Commun..

[35] Yang M., Guo D., Huang Y., Duong T.Q., Zhang B. (2016). Physical layer security with threshold-based multiuser scheduling in multi-antenna wireless networks. IEEE Trans. Commun..

[36] Zhang J., Pan G., Xie Y. (2018). Secrecy analysis of wireless-powered multi-antenna relaying system with nonlinear energy harvests and imperfect CSI. IEEE Trans. Green Commun. Netw..

[37] Wang D., Negra R. Design of a dual-band rectifier for wireless power transmission. Proceedings of the 2013 IEEE Wireless Power Transfer (WPT).

[38] Le T., Mayaram K., Fiez T. (2008). Efficient far-field radio frequency energy harvesting for passively powered sensor networks. IEEE J. Solid-State Circuits.

[39] Boshkovska E., Ng D.W.K., Dai L., Schober R. (2018). Power-efficient and secure WPCNs with hardware impairments and non-linear EH circuit. IEEE Trans. Commun..

[40] Clerckx B., Zhang R., Schober R., Ng D.W.K., Kim D.I., Poor H.V. (2019). Fundamentals of wireless information and power transfer: From RF energy harvester models to signal and system designs. IEEE J. Sel. Areas Commun..

[41] Niu H., Guo D., Huang Y., Zhang B. (2017). Robust energy efficiency optimization for secure MIMO SWIPT systems with non-linear EH models. IEEE Commun. Lett..

[42] Fan L., Lei X., Yang N., Duong T.Q., Karagiannidis G.K. (2017). Secrecy cooperative nerworks with outdated relay selection over correlated fading channels. IEEE Trans. Veh. Technol..

[43] Chen Z., Hadley L., Ding Z., Dai X. (2017). Improving secrecy performance of a wirelessly powered network. IEEE Trans. Commun..

[44] Mekikis P.-V., Antonopoulos A., Kartsakli E., Lalos A.S., Alonso L., Verikoukis C. (2016). Information Exchange in Randomly Deployed Dense WSNs with Wireless Energy Harvesting Capabilities. IEEE Trans. Wirel. Commun..

[45] Paing T., Shin J., Zane R., Popovic Z. (2008). Resistor emulation approach to low-power RF energy harvesting. IEEE Trans. Power Electron..

[46] Zhou X., Zhang R., Ho C.K. (2013). Wireless information and power transfer: Architecture design and rate-energy tradeoff. IEEE Trans. Commun..

[47] Shinohara N. (2014). Wireless Power Transfer via Radiowaves.

[48] Pham B.L., Pham A.-V. Triple bands antenna and high efficiency rectifier design for RF energy harvesting at 900, 1900 and 2400 MHz. Proceedings of the 2013 IEEE MTT-S International Microwave Symposium Digest (MTT).

[49] Kang X., Liang Y., Yang J. (2018). A new spectrum sharing paradigm for wireless-powered IoT devices. IEEE Trans. Wirel. Commun..

[50] Huang Y., Al-Qahtani F.S., Duong T.Q., Wang J. (2015). Secure transmission in MIMO wiretap channels using general-order transmit antenna selection with outdated CSI. IEEE Trans. Commun..

[51] Li M., Yin H., Huang Y., Wang Y. (2017). Impact of correlated fading channels on cognitive relay networks with generalized relay selection. IEEE Access.

[52] Dong Y., Hossain M.J., Cheng J. (2016). Performance of wireless powered amplify and forward relaying over Nakagamim fading channels with nonlinear energy harvester. IEEE Wirel. Commun. Lett..

[53] Boshkovska E., Ng D.W.K., Zlatanov N., Schober R. (2015). Practical non-linear energy harvesting model and resource allocation for SWIPT systems. IEEE Wirel. Commun. Lett..

[54] Gradshteyn I.S., Ryzhik I.M. (2007). Table of Integrals, Series, and Products.

[55] Do N.T., Costa D.B.D., Duong T.Q., Ng V., Bao Q., An B. (2017). Exploiting direct links in multiuser multirelay SWIPT cooperative networks with opportunistic scheduling. IEEE Trans. Wirel. Commun..

